# Special Issue “Function and Structure of Viral Ribonucleoproteins Complexes”

**DOI:** 10.3390/v12121355

**Published:** 2020-11-26

**Authors:** Serena Bernacchi

**Affiliations:** Architecture et Réactivité de l’ARN-CNRS UPR 9002, Université de Strasbourg, Institut de Biologie Moléculaire et Cellulaire, 67084 Strasbourg, France; s.bernacchi@ibmc-cnrs.unistra.fr

RNA viruses are extraordinary evolution machines that efficiently ensure their replication by taking advantage of the association with viral and cellular components to form ribonucleic complexes (vRNPs). These vRNPs are functional units of the infectious cycle, driving various processes including transcription, nuclear export, translation, and intracellular trafficking pathways for targeting viral components to the assembly sites where the packaging of the viral RNA genetic material into virions occurs. The aim of this Special Issue of *Viruses* is to provide an updated picture of the structural organization and of the different roles of vRNPs in various viral systems.

The first part of the SI is dedicated to HIV-1 which is the causative agent of AIDS. Since its discovery in 1983, HIV-1 has become one of the leading causes of death worldwide. Up to now, the pandemic persists despite the implementation of highly active antiretroviral therapy, and over the years, a vast spectrum of techniques has been implemented to diagnose and monitor AIDS progression. Besides the conventional approaches, recently, microfluidics has provided useful methods for monitoring HIV-1 infection. This powerful tool allows information at the single-cell scale and high-throughput development. Herein, Eid et al. highlighted recent significant advances of continuous microfluidics in AIDS diagnosis, and in the basic study of the HIV-1 life cycle [1]. The assembly of the HIV-1 virus progeny is orchestrated by the retroviral scaffolding polyprotein Gag (Group-specific antigen) which selects the viral genomic RNA (gRNA) for encapsidation into virions through specific interactions occurring between its nucleocapsid (NC) domain in its C-terminus and the packaging signal located at the 5’UTR of gRNA. To date, the initial Gag-gRNA interactions leading the vRNP formation are thought to occur in the cytoplasm, and vRNPs are then supposed to reach the plasma membrane where budding follows. In addition to the NC domain, the C-terminal extremity of Gag also includes the p1 (also named SP2) linker and the unstructured p6 domain which bind cellular and viral factors that are known to contribute to the last steps of the viral assembly. Immediately after or during budding, the virion undergoes a maturation process in which the viral protease subsequently liberates C-terminal maturation products of Gag that are NCp15 (NC-p1-p6), NCp9 (NC-p1) and NCp7. Tisné and collaborators carefully summarized the RNA binding properties of those NC proteins observing that the interactions between p1–p6 and the zinc fingers in the NC domain, can finely tune the NC-nucleic acids binding properties during the assembly [2].

Other retroviral Gag precursors as the Gag proteins of the Rous Sarcome Virus (RSV) are known to localize in the nucleus and form vRNPs complexes with the gRNA at that site. Although it seems that also HIV-1 Gag proteins were previously observed in the nucleus, so far, little is known about the role of their potential nuclear trafficking. Interestingly, Parent and co-workers [3] observed specific interactions occurring between HIV-1 Gag and gRNA in discrete foci in the nuclei of HeLa cells and detected Gag-gRNA at the perichromatin space in association with the regulatory HIV-1 protein Rev in a tripartite RNP complex. Importantly in latently infected CD4+ T cells, Gag was found to localize at the HIV-1 transcriptional burst site, forming vRNPs in the nucleus thus suggesting new sites for the occurrence of HIV-1 Gag-gRNA interactions. Indeed, the full achievement of HIV-1 viral assembly occurs through a set of interactions occurring between Gag and a variety of different biomolecules, including not only nucleic acids, but also lipids, viral and cellular proteins. The global map of human proteins involved in HIV infection was established due to technological improvements. Boutant and co-workers exhaustively summarized the putative functions in the HIV-1 replication cycle of the several interactions occurring between Gag and host cell factors, with particular attention to the interactions involving the NC domain of Gag [4]. Among the cell factors that take part in the HIV-1 life cycle, the mitochondrial lysyl-tRNA synthetase (mLysRS) is an enzyme that catalyzes the aminoacylation of the tRNA^Lys,3^. This last one serves as a primer for initiation of HIV-1 reverse transcription by its annealing to the primer binding site (PBS) in the gRNA, and is thought to be encapsidated into newly formed viral particles, through the interactions occurring between mLysRS and the viral precursors Gag and GagPol. Mirande and co-workers herein identified and characterized the amino acid residues located at the surface of the catalytic domain of mLysRS and in the IN domain of GagPol regulating the interactions of these two factors. This biochemical analysis contributed to suggest that the IN domain in turn stabilizes the tRNA^Lys^- mLysRS interactions [5]. Besides the well-established role of the maturation products of GagPol, which are the HIV-1 enzymes protease (PR), reverse transcriptase (RT), and IN (integrase), recent findings unveiled that IN would have an additional function involving its binding to the viral RNA genome in virions. This vRNP is believed to be necessary for proper virion maturation and morphogenesis. Indeed, the inhibition of IN binding to gRNA results in mislocalization of the viral genome inside the viral particles. In this SI, Elliott and Kutluay described both functions of the IN enzyme highlighting the common features of these roles, and describing how the IN binding to the gRNA is coordinated by the major viral structural protein Gag [6]. Similarly, to HIV-1 Gag, many other proteins displaying RNA chaperone activities encoded by viruses drive different steps of the viral cycle. This is the case of the small Heptaitis B Virus (HBV) Core protein, and in their review, de Rocquigny et al. discussed the many roles of the distinct domains of this protein that structurally contributes to the formation of capsid shells, drives the genome release in the nucleus, and ensures the protection of the viral genome [7]. Interestingly, the phosphorylation of the C-terminal domain of HBc regulates its interaction with nucleic acids during the assembly and maturation of HBV particles.

Among the RNA viruses, the Orthomyxoviruses and Retroviruses display replicative stages in nuclei. The access to the nucleus for the initial nuclear import of the viral genome, the nuclear export of viral RNAs for translation, and the nucleo-cytoplasmic shuttling of the viral proteins constitute complex processes that require the participation of many proteins that need to be recruited. In this context, Dimitrova and collaborators focused on the advances made over the last few decades in studies on viral mRNA nuclear export mechanisms and presented detailed insights into the most important strategies that viruses use to export their RNAs from the nucleus to the cytoplasm to complete their replication cycle [8]. Moreover, the influenza viruses are negative single-stranded RNA viruses with nuclear transcription and replication, and they enter the nucleus by using the cellular importin-α/-β machinery. Viral nucleoproteins from influenza A, B, C and D viruses possess a nuclear localization signal (NLS) localized on an intrinsically disordered extremity of nucleoproteins (NP_TAIL_). In their work, Crepin and collaborators provided the first comparative study to dissect the interactions between the four NP_TAILs_ and the four importins-α identified and provided important highlights of these complex translocation mechanisms [9].

The structural conformations of vRNP components also play a crucial role in the formation of the complexes and in their stability. This is the case of the nucleoprotein (NP) of the negative strand RNA Arenavirus, which features a globular RNA binding domain (NP-core), and plays major roles in the viral life cycle, by ensuring the defense of the gRNA in the cytoplasm and by interfering with the immune system. Interestingly, NP conformational changes are necessary to ensure efficient viral RNA encapsidation, and in their review, Ferron and co-workers revisited the most recent functional implications and structural data available on Arenavirus NPs [10]. Further, in the Rhabdovirus context, Riedel et al. explored the higher-ordered structural features of vRNPs. Indeed, the viral particles of these single-stranded negative-sense RNA viruses are bullet-shaped and contain a helical RNP consisting of highly structurally conserved building blocks [11]. Moreover, the role of secondary structural elements of the viral RNA in the formation of vRNP is exposed in the work of Andreoletti and collaborators [12]. In effect, the 5’ of the viral RNA of the Group-B enteroviruses (EV-B) contains a highly structured domain-I that interacts with viral or cellular proteins to form vRNPs which regulate the viral replication.

Moreover, vRNP can also contribute to coordinate the gene expression of viruses as the human papillomaviruses (HPVs), since complexes consisting of viral pre-mRNAs and multiple cellular RNA-binding proteins were found to control different aspects of gene expression, and contribute to the progression of the life cycle and HPV-associated cancer development. In their review, Kajitani and Schwartz summarized the regulation of HPV16 gene expression at the level of RNA processing with a focus on the interactions between HPV16 pre-mRNAs and cellular RNA-binding factors [13]. Finally, among plant viruses, the tomato bushy stunt virus (TBSV) is one of the best-studied plant viruses since its host range covers a wide spectrum of plants. Ritzenthaler and collaborators identified and characterized a TBSV isolate that efficiently infects Arabidopsis [14]. This isolate provides a new model for dissecting plant–virus interactions and was shown to replicate in association with clustered peroxisomes. Furthermore, host dsRNA-binding proteins, associated with TBSV viral replication complexes (VRCs) were localized and identified.

I do hope that this Special Issue of Function and Structure of Viral Ribonucleoproteins Complexes provides substantial new information on vRNPs in various viral systems, and fosters novel research to better understand the molecular organization of those complexes, as well as their associated implications in the mechanisms promoting the RNA virus machine replication.
